# Impact of COVID-19 lockdown and link to women and children’s experiences of violence in the home in South Africa

**DOI:** 10.1186/s12889-022-13422-3

**Published:** 2022-05-21

**Authors:** P Mahlangu, A Gibbs, N Shai, M Machisa, N Nunze, Y Sikweyiya

**Affiliations:** 1 Gender & Health Research Unit, South African Medical Research Council, Private Bag X385, 0001 Pretoria, South Africa; 2 School of Public Health, University of the Witwatersrand, Johannesburg, South Africa; 3 Centre for Rural Health, School of Nursing and Public Health, University of KwaZulu Natal, Pietermaritzburg, South Africa

**Keywords:** Intimate partner violence, Domestic violence, COVID-19 impact, Lockdown, Women, Children, South Africa

## Abstract

**Background:**

Evidence on the impact of COVID-19 and lockdown remains at an early stage. There is limited research about the impact of hard lockdown restrictions on families, specifically how these restrictions impact on women and children’s experiences of domestic violence, including intimate partner violence (IPV) and child abuse in South Africa. We conducted research among men and women in Gauteng province, South Africa to understand their experiences of the COVID-19 national lockdown and its impact and link to women and children’s experiences of domestic violence.

**Methods:**

We conducted a qualitative study, using social media to recruit men and women who were 18 years and older, living with a spouse and/or children in Gauteng province, South Africa during the lockdown. To collect the data, we conducted telephone interviews, and analyzed data using the thematic approach.

**Results:**

The lockdown had unprecedented negative economic impacts on families, and exacerbated some of the risk factors for violence against women and children in the home in South Africa. Some women reported experiences of emotional violence. Experiences of physical violence were mostly amongst children. The risk factors for women and children’s experiences of violence in the home differed by socio-economic class. Job losses and reduction in earnings resulted to food insecurity which was a key driver of violence in most low socio-economic status (SES) families. Confinement in the home with spouses was an unfamiliar and difficult experience, associated with conflict and perpetration of violence by men in high SES families. Participants across socio-economic groups reported high levels of stress with limited psychosocial support available during the lockdown.

**Conclusions:**

Our finding showing a link between low-socio-economic status and increased risk for domestic violence during the lockdown in South Africa suggests the need for socio-economic interventions to mitigate these risks. Structural and social relief measures need to be strengthened to reduce the loss of jobs and income and to address food insecurity during pandemics. Psychosocial support should be provided to men and women to mitigate the mental health impacts of the pandemics and lockdown.

## Introduction

Globally, many countries, including South Africa are battling the Coronavirus (COVID-19) pandemic, and implementing measures to contain the spread of the virus. South Africa reported the first domestic case of COVID-19 on the 4th of March 2020. On the night of the 23rd of March 2020, the South African government announced a 21-day national lockdown known as “Alert level 5” to come into effect from 26 March to 16 April 2020. The total number of confirmed COVID-19 cases in South Africa at the time was 402, of which 207 (51%) were recorded in Gauteng. These numbers indicated a rapid increase of 128 cases from the previous day. On the first day of the national lockdown, South Africa recorded the first death due to COVID-19 virus, and the total number of COVID-19 confirmed cases was 1170. Alert level 5 was further extended until 30th of April 2020, followed by four subsequent alert levels which involved the gradual easing of restrictions, one level at the time between 1st of May – 27 December 2020, as the number of COVID-19 positive cases went down [1, 2]. The five alert levels were classified based on the level of infections and rate of transmission, the capacity and readiness of the health system, the extent of implementation of the public health interventions, and assessment of the social and economic impact of continued restriction [2]. Alert level 1 indicated a low COVID-19 spread with high health systems readiness [2].

During Alert level 5, described in this paper as the hard lockdown, indicative of high COVID-19 spread with a low health system readiness, people were required to stay at home, schools were closed, and people were not allowed to go outside of the home for exercise, or any other reason, beyond seeking or providing essential services and food. Interprovincial and international travel was banned [1]. This meant that many parents stayed at home with children, with some parents working from home, and some children doing online schooling. Families found themselves in unusual situations. Research suggests that in some families traditional gender roles were more observable, women were more involved in domestic chores and family care, while men made decisions about finances and grocery shopping [3, 4]. For some, research suggests the COVID-19 lockdown challenged traditional gender roles and beliefs about ‘being a man’ in the family, with men less likely to perform ‘traditionally’ masculine behaviors including work outside the home due to lockdown restrictions [5]. This primarily impacted men who lost earning power and were not able to provide and fulfill the ‘breadwinner’ role, and those who experienced shifts around division of house work [6, 7].

There remains limited research about the impact of hard lockdown restrictions – such as South Africa’s Alert level 5 - on families, specifically how these restrictions impact on domestic violence, which is violence that occurs in the home, including intimate partner violence (IPV) and child abuse, or maltreatment. However, emerging evidence globally and in South Africa suggests that vulnerability to domestic violence was exacerbated during the COVID-19 lockdown [8–10], and that pandemics more generally exacerbate violence against women (VAW) and violence against children (VAC) [8, 11, 12].

There are a number of reasons why domestic violence may have increased during COVID-19 lockdown. When hard lockdowns are in force, women and children are confined in the home and temporarily restricted from escaping abusive partners or perpetrators, which increases their risk of experiencing violence in the home [13]. Furthermore, isolation and prolonged confinement in the home is linked to poorer mental health and increased substance use, which are associated with an increased risk for VAW and VAC perpetration and experience [14, 15]. During hard lockdowns many people are not able to work, or earn an income, resulting in increased economic insecurity and a lack of basic necessities (e.g. food), which can increase conflict over resources in intimate relationships [11, 16]. Economic insecurity during lockdown is also associated with increased stress which might contribute to conflict in the home [17, 18]. Concurrent remote working and caring for children who are out of school due to COVID-19 lockdown is stressful for caregivers and exacerbates risk of domestic violence including child maltreatment [19, 20].

In South Africa there is limited evidence about the impact of COVID-19 lockdown on women and children’s experiences of violence in the home, although there is evidence emerging about impact on livelihoods. This data would be useful to inform the development of prevention and response guidelines to implement in future pandemics, particularly in the context of high level of VAW and VAC in South Africa [21, 22].

One in 4 adult women in the general population in South Africa have experienced gender-based violence (GBV) [23, 24]. Half of women (51.3%) who participated in a population based study on GBV in Gauteng Province in South Africa, have experienced GBV in their lifetime. Most women (43.7%) had experienced emotional violence, and 37.7% have ever experienced physical and/or sexual intimate partner violence [23]. In South Africa, a national prevalence study estimated that 1 in 3 children have been victims of sexual violence and physical abuse before they reach age 18, 12% experienced neglect, and 16% reported emotional abuse [25].

To contribute to the growing body of evidence, we interviewed men and women about their experiences of the COVID-19 national lockdown, and its impact and link to women and children’s experiences of violence in families, in Gauteng province, South Africa. We were interested to explore all forms of violence which might have occurred in the home, including verbal, physical, psychological, sexual, and economic violence, against women and children, perpetrated by an intimate partner, parents, siblings, or family relatives during lockdown. Both men and women who experienced conflict in the home were asked to share strategies used to manage conflict during lockdown.

## Methods

We conducted an exploratory qualitative study to explore women and men’s experiences of the COVID-19 pandemic and lockdown, and its association to perpetration and experiences of domestic violence, specifically targeted towards women and children. The study was conducted in Gauteng province, a province with the highest number of COVID-19 cases in South Africa.

We asked women about their experiences of the lockdown and how it impacted on family relations including relationship with intimate partners and children in the home. Women were asked to reflect on the perceived impact of COVID-19 and the lockdown on their livelihood, food security, mothering, mental health (stress) and coping in the home. We further explored amongst women whether they or their children have experienced violence during lockdown, and those who reported the experience of violence were asked to describe the form, context and nature of their violent experiences in the home during lockdown. We asked men about their experiences of the lockdown and how it impacted on family relations including relationship with intimate partners and children in the home. Men were also asked to reflect on the perceived impact of COVID-19 and the lockdown on their livelihood, ability to provide, fathering, mental health (stress) and coping in the home. Both men and women who reported experience of conflict in the home were asked to share strategies they used to manage it during lockdown.

### Recruitment and data collection

Participants were recruited using Facebook and WhatsApp. About 88.2% of the population in Gauteng have access to functional cellular (mobile) phones, and 73.1% have access to internet and use social media platforms (including Facebook and WhatsApp) using mobile devices [26].The study advert was posted on the South African Medical Research Council’s (SAMRC’s) Facebook page and shared by the study team on their personal Facebook accounts, inviting anyone who was eligible to participate. The advert was also shared by the research team with social networks via WhatsApp. We encouraged social networks to share the advert further. The study advert had an email address and a cellphone number, which prospective participants used to contact the study team if they were interested to participate. When a prospective participant contacted the team via a WhatsApp message or email, we contacted them to further explain the study, screen for eligibility, and to take them through an informed consenting process.

Study eligibility was women or men aged 18 years and older, residing in Gauteng for an uninterrupted period between February and July 2020 during the lockdown, living with a spouse and/or child(ren). In addition, we asked participants to indicate their income bracket and used the Statistic South Africa annual household income classification to categorise participants into low-income, or middle and high-income groups [27]. The need to have privacy when conducting interviews was explained to participants and those who indicated that they had a private space to conduct the interview were included in the study. Interviews were scheduled based on participants’ preferred day and time. Participants gave written informed consent using WhatsApp, text and email.

The study targeted to recruit and collect data from a total of 40 participants (20 women and 20 men). In-depth telephonic interviews were conducted with 37 adult men and women. However, by interview 35, no new information was forthcoming, and after two additional interviews the authors determined they had reached data saturation. In total we had 19 adult women aged 33 to 59 (10 low income and 9 middle – high income) and 18 men aged 24 to 62 (10 low-income and 8 middle and high income). Thirty interviews were conducted during alert level 3, and five interviews during alert level 2, and two during alert level 1, covering the period from July 2020 – September 2020. Telephone interviews were conducted to comply with the COVID-19 social distancing measures to protect both the researchers and the participants. A semi-structured interview guide with open ended questions was used to conduct interviews by the research team matched by gender and in the language chosen by the participants. Most interviews were conducted in English, and a few in IsiZulu, IsiXhosa, or SeSotho.

Ethics approval for the study was granted by the SAMRC Human Research Ethics Committee, and the research adhered to the WHO’s ethical standards for conducting VAWC research, ensuring privacy, participant safety during interviews, and referral to services information was made available to participants. Participants were reimbursed R100 (7.13USD) sent on a digital e-wallet.

### Data analysis

All audio-recorded interviews were transcribed verbatim and those in other languages were translated into English by a research assistant. Data were firstly analysed inductively using thematic analysis [28]. Themes were generated through the analysis process. Thereafter, there were deductive elements to our analysis. We specifically focused on the themes related to women and children’s violence experiences and its drivers in the home. Transcripts were read repeatedly, and initial codes developed based on the interview guide and phrases representing segments of the text in the transcript. Codes were manually developed using MS Word. The codes were used to develop a codebook. Following this stage, the applicability of the codebook using the raw data from the transcripts was reviewed and tested, which led to an expansion of codes. Text which seemed to fit together was grouped together under a specific code [29]. Further to this, data were explored and numerous open codes were identified. Similar open codes were grouped together under defined categories [29]. Lastly, relationships between the categories were explored, and emerging themes drawn from the data [28].

## Results

Four main themes emerged during data analysis. First, participants’ accounts provided insights on women and men’s experiences of the COVID-19 lockdown and how it impacted and links to women and children’s experiences of violence in the home during lockdown. Second, our analysis revealed key drivers of violence experienced by women and children, perpetrated by men during lockdown. Third, the analysis illustrated the differential impact of hard lockdown on the participants based on their socio-economic status. Last, our analysis illuminated the different strategies employed by our participants in managing conflict and violence in the home during lockdown, which have important implications for prevention of domestic violence in future pandemics.

### Women and children’s experiences of violence during COVID-19 lockdown

Our data revealed that some women experienced violence during the hard lockdown, primarily emotional partner violence including shouting, insults and manipulation. Only two of the 19 women reported experiencing physical partner violence during lockdown. There were also reports of physical violence against children during lockdown.

#### Emotional violence experienced by women and children in the home

Experiences of emotional violence were mostly reported by women in the low SES bracket, with relatively few reports from women in the high SES bracket during lockdown. Emotional violence against women was primarily perpetrated by their male spouses and intimate partners. Amongst women in the low SES, emotional violence was described as chiefly caused by a lack of food and other necessities in the home. Among women in the high SES bracket, confinement in the home was associated with increased stress and aggression from spouses during lockdown. Mapula, from the low SES bracket, described that while she did not experience physical violence, she had to live with a frustrated and angry partner who had lost income during the lockdown.

*“My husband was working for a company which was affected by ban of alcohol sales. They did not get paid if they did not work … We had fights because there is lack of income in the house. We don’t fight physically, he gets harsh when he responds. It is more the verbal exchanges, angry responses, and a cold-shoulder after arguments, but not physical…”* (Mapula, woman, low SES).

Some participants spoke about children witnessing outbursts of the emotional violence in the home during lockdown. A female participant shared her experience:

*“We are fine. We once argued and said bad things to each other, shouting while my children were around…The older one came to speak to us, telling us to stop but we continued because we were both angry. The younger child was crying, everyone was at home*.” (Mpumi, woman, low SES).

Some parents described staying at home during lockdown as stressful and that it made them become cranky and aggressive towards children. Mathapelo explained her experience of increased stress and short-temper because she could not work, during the hard lockdown:

*“I`ve become very aggressive and there was a time I cried because I hit him [son]. Because of the stress I get irritable, I shout and sometimes hit my 4-year-old son, he is naughty, likes attention and I always lose my temper around him. I can’t wait for him to go back to school… Ja…[crying].”* (Mathapelo, woman, low SES).

Many participants reported having to make budgetary adjustments because of less income, and this had a negative impact on their relationships with spouses at home. Some reported increased conflict, which resulted from having to cut down on spending and having limited resources and food during lockdown. For Lucy, a single parent, her efforts to ensure she saved money by eating less strained her relationship with her son. She regretted her impatience and saying things she did not mean to her child:

*“We had a number of fights with my elder son, he wastes food now that he is home, while as a single parent I am trying to save… He is a very stubborn person who backchats when you speak. Talking nicely with him does not work. I try not to beat him, but I lose my temper and say things I regret when I am calm. I do not like that because he sometimes feels I do not love him, and only love his siblings*.” (Lucy, woman, high SES).

Mukundi similarly described how tough decisions about how to manage the reduced family budget caused arguments between him and his spouse:

*“We argued a lot over reducing how we spend the less salary I was now getting and that affected our relationship. She never understood [why they needed to cut spending], and I had to work-hard to show the person this is how we will now live in this house. I would apologise for my tone, but still make my point… It was very challenging to convince someone to come to their senses to understand that this is not the same time like before [the hard lockdown]*” (Mukundi, man, low SES).

### Key drivers of violence experienced by women and children during COVID-19 lockdown

Our data suggested that the drivers of violence against women and children in the home were diverse, and partly contingent on the socio-economic status of families. Food insecurity was most prominent in influencing the stress, arguments and conflict reported in the low SES households, while among high SES families, descriptions focused more on how confinement at home during lockdown resulted to stress and contributed to experiences of violence.

### Food and basic provision

Most participants in the low SES group had either lost their jobs, had their salaries cut, or their livelihood strategies were no longer viable during lockdown. As such, they struggled to buy food, and experienced extreme food-insecurity. Lack of access to food was a source of tension, arguments and conflict amongst the low SES group. Our data highlighted how despite many men losing income or earning less during the lockdown, there remained a significant gendered expectation that men should continue to provide economically for their families, while women had to use whatever resources they were given to take care of the household and prepare food. Some men, who did not have money felt challenged and responded in ways that were described as emotionally abusive when they were asked to provide.

Mpumi lived with her husband whose source of income was a mini-bus (a common form of public transport) he used to transport children to school. However, with schools closed during the hard lockdown, he could not transport children. Mpumi described how the lack of money and not being able to buy food, led to arguments in the home. She felt that her husband was shifting responsibility to provide, and that she was the only one trying to ‘make a plan’ for them to have food:

*“It was not a nice fight because we said things that we can never take back and it’s painful…uhm… My husband is a quiet person. When we do not have food, I am the one who has to see and make a plan. I told him that day that he needs to make a plan. He just stood and shouted that I do not understand…where do I think he gets the money and all that… Nothing happened we were arguing, I was very upset and crying because I felt he is being irresponsible and not taking care of us as he should and he was not understanding what I meant*.” (Mpumi, woman, low SES).

Thabo, who lived with his unemployed spouse, received half his salary during the hard lockdown period as he worked three, instead of six days a week. He described the negative impact of the salary-cut in the family, feeling blamed and pressurised by his spouse to continue to provide even when he was unable:

*“Like I said, I am working in a food store, my wife does not work. Sometimes when I get home I find that there is no food. Sometimes she would blame me and pressurise me. We end up having a misunderstanding and fighting. She would say “you are the father you should make a plan” Sometimes we used to fight physically and break things in the house, and sometimes verbally I was unstable when it comes to providing food, that is where the problem was and we ended up fighting. It was a situation where there was nothing in the house, then it was where we were fighting because she was pressurizing me to make a plan*.” (Thabo, man, low SES).

Precious’ husband stayed at home for four months, and was paid 40% of his salary, a situation that he struggled to accept. Precious described how her husband did not want to talk about anything related to the salary-cut, and whenever she raised the issue of food with him, he would become aggressive:

*“I always knew and had to prepare myself before asking what we going to eat for dinner. His answers are very hurtful, and he feels I am attacking him, that always brings arguments between us*.” (Precious, woman, low SES).

Similarly, Mapula described how her male partner, who was retrenched from his job during the hard lockdown, refused to engage on discussions about what they were going to eat, and rather perceived that his manhood was questioned because he no longer has money:

*“When I ask him what are we going to eat, he is like, ‘just because I don’t have money now it’s a big thing’, and I am like because he as the father, must figure out what we are going to eat*.” (Mapula, woman, low SES).

Some men also expressed that not being able to provide for their families during the hard lockdown made them feel less of a man, and this affected them emotionally:

*“I feel bad and frustrated as a father. As the leader of the house you must provide for your kids, because if the kids look to me and say we are hungry, and my wife says oh the kids are hungry, I am the one who is responsible to provide in the family. Yet on the other side, I am struggling and there is nothing I can do with it, it makes me angry. I feel like I am not man enough, though I was trying to get a little from somewhere, you understand, it has affected me very badly*.” (Vuyo, man, low SES).

Similarly, Rhulani described the anger he felt as a man because he could not provide for his family:

*“As a man I was feeling like I am not responsible and not man enough, she [spouse] used to ask me, ‘so now where are we going to get the food’? For me if I cannot provide for my family I am not man enough. It made me angry*.” (Rhulani, man, low SES).

### Stress of being confined together at home

For some participants, spending time at home strengthened bonds between parents and children and between spouses in the early days of the lockdown, particularly in families where spouses usually spend most of their time at work. However, many participants reported increased stress during lockdown. The causes of stress were different between the two income groups. Participants in the high SES reported increased stress due to confinement at home, particularly when movement and outdoor time was restricted in level 5 (hard lockdown) and them having to work remotely. Most participants in the low SES spoke about stress caused by loss of jobs and earning, worrying about survival, and meeting basic needs.

The continued lockdown and confinement in the home became increasingly difficult and many of the men interviewed found it difficult to adjust. Mthokozisi described his unfamiliar experience of having to spend more time with his spouse during lockdown as thus:

*“I had to stay in the house with my partner and that is something I was not used to doing. I knew that when she is at work I will be left alone in the house, and now we have to stay together full time, look at each other in the eyes, the whole six months!*” (Mthokozisi, man, low SES).

Some men said staying at home frustrated them and caused tension because they felt their female partner was not doing what they felt they should be doing in the home during lockdown. Vuyo explained that staying at home led to him commenting on a range of ‘small things’ his wife did, which triggered arguments. This pettiness, he argued, had an impact on his self-esteem:

*“So, the way that I have seen [experienced] it, the lockdown killed us and killed the self-esteem on men because most of the time when you are at work you do not argue with your wife, but when you are together for a long time there are things you see that you do not see when you are at work. You react when she does not do things the way you would want her to do them*.” (Vuyo, man, low SES).

Further demonstrating men’s expectations on which housechores their female partners should be doing, David spoke about his frustration when he felt his wife was lazy and not doing what he expected of her:

*“For us, the tension, and arguments we had was when I felt my partner was lazy. While she could be cleaning and cooking or doing something, she would be lying in bed for example*.” (David, man, high SES).

### Children did not want to be cooped up

During the hard lockdown period schools were closed, and slowly reopened from 1st of June 2020, during Alert level 3. Participants, mainly from low-income families, described the challenges they faced in keeping children entertained in the home during lockdown. Some described how the restrictions did not consider the living conditions and circumstances of low-income families with limited space and less options to keep children entertained while at home:

*“They didn’t consider our houses here in area X, which are small with limited space. How will Ndumiso learn to ride a bicycle here? The houses they [media] were showing us [on television], where families and children would be able to stay at home have gardens, many rooms,and there are groceries in that kitchen, you understand?”* (Jeffrey, man, low SES).

Similarly, other participants from the low SES group described how they struggled to stop children from trying to go out and play with their friends. Parents would yell, shout and hit children, as they tried to keep them at home to protect them from acquiring COVID-19.

“*You know kids are very naughty, so sometimes I had to be harsh on them so that they can cooperate. I used to be harsh when I spoke my children… Example is when I locked the gate and the kids climb the gate to go out, I would shout and beat them, then they started to behave.”* (Thabo, man, low SES).

One male participant who was very concerned about ensuring his teenage nephews did not contract COVID-19 sought to lock them in the house, yet this almost led to physical fights and strained their relationship:

*“In terms of the relationship, it was affected negatively because I became harsh to my nephews trying to stop them from going out and our relationship became very bad.I have decided to just leave them because they go out more than five times a day and in terms of our relationship with them it has stopped [was negatively affected]… I started to lock the gate, they became angry and telling me they want to go, and I said you can’t go there and then the older one wanted to fight me physically, saying ‘we cannot be locked in this house forever’.”* (Tebogo, man, low SES).

Keeping children occupied during lockdown was not such a challenge for most participants in high-income families. Children had various options including playing in the garden, electronic gadgets, online schooling and games. Rather most participants in high SES families struggled with having to balance working from home, looking after small children, and providing emotional support to their children:

*"Well it is very hard because you need to prepare, you need to cook three times a day, make sure there is breakfast, lunch, clean up for everyone in between, keep an eye on children, and must be in zoom meetings for work."* (Paul, man, high SES)

### ‘People were left with nothing’: lack of psychosocial support

Closure of services providing psychosocial support and isolation from social networks during lockdown left people with limited coping strategies during lockdown. Mthokozisi for instance would normally go out with his friends on weekends as a way of coping with daily life stresses:

*“I used to go out with friends on weekends, but because of the pandemic most of the places were closed and sometimes we couldn’t find something to drink, so life has changed, making it difficult for one to cope with all the stresses.”* (Mthokozisi, man, low SES).

Similarly, Thabo described how he could not go to his church, where he received social and spiritual support and guidance:

*“If I was able to go to church I think things would be easy for me and I would get support… The lockdown as a whole has affected me a lot spiritually.”* (Thabo, man, low SES).

### Strategies used to manage conflict and violence in the home

Most participants said they struggled to manage conflict and arguments in the home during lockdown. Several participants got support from family members:

*“We decided that we should call [family] elders to come and advise us, because the conflicts will never take us anywhere. So as for us, I have spoken to her about why we are always fighting, as before [lockdown] we were not like this. She said she doesn’t know and I said we should call the elders to come and advise us on how to deal with this situation [fighting]. We ended up agreeing, so her granny and one member of my family sat us down and we tried to find the root of the problem.”* (Thabo, man, low SES).

Others described using self-calming strategies to deal with the stress of lockdown. This included being calm, sensitive and apologising to spouses when wrong:

*“How I resolved it, I used ‘self-therapy’, I just became patient with her… if I find that whatever I said didn’t sit well with her, I would apologise for the way I spoke…also still sticking to the very same comment to say, this is what I was trying to say that if the way that I speak, or my tone was not okay, I withdraw that. I used my calm language so that I can be able to find a solution.”* (Mukundi, man, low SES).

## Discussion

This study explored the impact of the COVID-19 pandemic and hard lockdowns and its link to women’s and children’s experiences of domestic violence in the home in Gauteng, South Africa. We have showed contrasting experiences of lockdown by gender and social class. Our findings highlight how men and women were differently impacted by the lockdown, and how that became a risk factor for violence experience and perpetration in the home in the low and high SES groups. Moreover, our findings have shown that conflict in intimate relationships and emotional violence against women in the home was common during lockdown Alert level 5 in South Africa, mostly reported by women in the low SES. Some participants, mainly women, further reported perpetration of physical violence against their children during hard lockdown. Of note, is that very few women reported experiencing physical violence in our study, despite studies globally indicating a rise in physical violence against women during lockdowns [30].

Our findings suggest that the hard lockdown exacerbated some of the known risk factors for VAW and VAC. Experiences of violence by women and children in the low SES families were linked to the negative economic impacts of the lockdown, which included pay-cuts, loss of jobs and income in families. Job losses were concentrated amongst the already disadvantaged, with women accounting two-thirds of the total number of job losses between February – April 2020 in South Africa [31, 32]. Similar to other studies, we found that increasing food insecurity was a key driver of violence in low SES families during lockdown in South Africa [33, 34]. Low SES participants reported food shortages and not having money to buy food as a result of job losses and less earnings during lockdown. The National Income Dynamics Study – Coronavirus Rapid Mobile Survey (NIDS-CRAM) wave 1 data showed that almost half (47%) of participants had run out of money to buy food in the month of April during the hard lockdown [35]. Other South African studies have shown similar findings about the impact on food security among low SES families during lockdown [6, 36]. Studies have shown that food insecurity has both a direct and an indirect association to IPV perpetration and experience through the pathways of increased stress, worsened mental health and reduced relationship quality [37–39]. While in most studies food insecurity is associated with women’s experience of physical and sexual IPV [40], our study highlighted a link between food insecurity and women’s experience of emotional violence. Structural and social relief interventions need to be strengthened to minimise food insecurity during pandemics. While structural interventions and food relief programmes were rolled-out in South Africa, challenges including inconsistent and insufficient supply were reported. Our findings suggest the need for a more robust approach to support low-income households who were most affected by the socio-economic impacts of COVID-19 and lockdown.

Our data further highlighted how loss of earnings and greater food insecurity increased the likelihood of violence through gendered expectations around the provision of food and the conflict this engendered. In this study, despite job losses and reduced income, men were still expected to provide food in the home. This increased men’s stress and men started to feel inadequate when they could not provide. Some men feeling pressured to provide, used physical and verbal aggression to deflect these feelings. Research conducted in Kenya’s slums show that even in circumstances of hardship, the expectation that men should provide remains strong [41]. There was often poor communication about the challenges households faced economically, quite possibly because of the overwhelming sense of lack of control people felt during this period. Some men were angered by the continued expectation to provide food and felt their masculinity was being questioned by their spouses. Some men expected their spouses to make a plan to feed the family when they could not provide food and this created confusion and resulted to uncomfortable verbal exchanges between spouses in the home. Aggressive behaviors were also reported in families where women’s views in decision making about dealing with the reduced income were not valued, and where men used verbal aggression to make their spouses ‘understand’ and adjust to the changed financial status.

Extant literature suggests that some men may use violence to regain a sense of control [42, 43]. In the context of the huge uncertainty over how the COVID-19 pandemic was going to play out, including the uncertainty about how severe the illness could be and the length of hard lockdown, alongside the removal of employment and earnings during lockdown, it may be that this contributed to violence perpetration by some men. Men might have used violence and control as a way to assert some control in their life and assert their authority [44, 45]. Aggression towards and suppression of female partners views when making decisions about the use of limited money in the household may have been one way of reasserting one’s manhood [46].

Our study also showed that the lockdown amplified the violence experienced by children in the home, and reflects findings seen in other contexts [19, 47]. Parents in both low and high SES families reported increased stress during lockdown. The finding about increased stress amongst parents during lockdown is consistent with other studies [48, 49], but the underlying causes were different by SES status. Among those in the high SES group, the COVID-19 pandemic and lockdown introduced external stressors including working remotely, not having domestic help, and home schooling of children. Balancing these responsibilities and demands was stressful for some parents. Job losses and reduced earnings were primary sources of stress among low SES families, with parents worrying about their survival and that of their families during the lockdown. Increased stress was associated with maltreatment of children (aggression, yelling and hitting) by some parents, mainly mothers. This finding reflect those of other studies which have shown that parents who experience stress are more likely to engage in child abuse and neglect [20, 50, 51].

Being confined at home with spouses was for some high SES men a difficult experience and a source of stress associated with conflict and arguments over trivial issues in the home. For these men, who usually spend their time at work or outside of their home with friends, staying at home with their spouses during lockdown was unusual and frustrating. During the lockdown men started noticing their partner’s failings and inability to do what they expected them to do, suggesting men’s need to seek control over women, which led to conflict in the home. Our findings are consistent with research showing that confinement stress impacted various aspects and strained relationships in the home [47, 51]. However, a few men described enjoying the additional time with their partner.

Many of the sources of support for families to deal with conflict and stress, including access to formal and social support through networks including family, churches and friends were not available during the lockdown. Without social support, many struggled to manage the increased stress during lockdown. While most described limited social support, a few described seeking support from their families when they were particularly pressured, suggesting that households are actively trying to mitigate the impact of the stresses of COVID-19.

### Limitations

This study had several limitations that should be considered when interpreting the results. Our analysis is drawn from a limited sample of men and women in Gauteng and not generalizable. Data were collected using telephone interviews which presented methodological challenges including limited ability for researchers to establish rapport and connection with participants. While telephone interviews are a convenient and safe data collection tool during pandemics, they presented limitations in our study as some participants were reluctant to disclose sensitive information. Women were reluctant to talk about personal experiences, and more willing to talk about physical violence experienced by friends, relatives and neighbours. There are a number of reasons that might have contributed to women not disclosing experience of physical violence during interviews. It may be that personal issues, such as experiencing violence are difficult to talk about over the phone [52], particularly to interviewers who are not known to participants. Additionally, some women may have been concerned about being judged negatively if they disclosed violence experiences [53, 54]. There is a likelihood of social desirability bias in our study. Violence (perpetration and experience) is sensitive and a socially undesirable behaviour, which might have been a contributing factor to underreporting by participants in our study. It is also possible that the less reporting of physical violence in our study could be an effect of recruitment bias within the study, whereby people in families that were less violent may have had greater affinity to participate than those in “more violent families”. However, we are not able to estimate the extent to which recruitment bias may have impacted our study. Data about children’s experiences of violence were reported by men and women who participated in our study and not the children. We did not interview children about violence during lockdown because of the ethical and methodological challenges. While this study mainly focused on women and children’s experiences, future studies would do well to also explore men’s experiences of violence during lockdown. Lastly, we did not have data about participants’ experiences of violence in the home before lockdown to be able to make comparisons about changes. We only drew from participants’ reports of experiences and perpetration during lockdown period.

## Conclusions

Our research has shown that the lockdown has had unprecedented negative economic impacts on families, and exacerbated some of the risk factors for VAW and VAC in the home. Our data revealed that some women experienced violence during the hard lockdown, primarily emotional partner violence. Reports of experiences of physical violence were mainly directed towards children. These findings highlight the need for governments to develop evidence-based strategies to prevent and respond to violence in the homes during lockdowns. When developing lockdown regulations, policymakers need to put in place evidence-based measures to prevent violence against women and children. Strategies need to include financial support to limit food insecurity in households. Provision of support services is also essential and should include improving access to safety for women and children experiencing violence in the home. Centres for victims of domestic violence need to be considered as essential services, to be operational during hard lockdown, but also ensure protection and safety of staff and users. Psychosocial support should be provided to men and women to mitigate mental health impacts of the pandemics, likely to undermine efforts to end violence against women and children. Future research is needed to further explore the impacts of pandemics like COVID-19 and the lockdown and its link to women and children’s experiences of violence in the home over a long-term. A survey, using a self-administered questionnaire with a bigger sample is warranted to allow for further exploration of the magnitude of domestic violence and its drivers, which was difficult to explore amongst participants over the phone.

## Data Availability

The datasets generated and used and/or analysed in the study are available from the corresponding author on reasonable request.

## References

[1] Republic of South Africa. Government Gazette. Department of Cooperative Governance and Traditional Affairs. Pretoria: Government printers. 2020. p. 1–16.

[2] Republic of South Africa. Disaster Management Act: Directions and criteria that will guide the determination of alert levels. Department of Health. Pretoria: Government printers. 2020. p. 1–3.

[3] Casale D, Posel D. Gender inequality and the COVID-19 crisis: Evidence from a large national survey during South Africa’s lockdown. Res Soc Stratification and Mobility. 2021;71(1):1–5. 10.1016/j.rssm.2020.100569 PMC9756129 36540167

[4] Parry BR, Gordon E (2021). The shadow pandemic: Inequitable gendered impacts of COVID-19 in South Africa. Gender, Work Org.

[5] Tahir MW, Störmer M, Zafar M. Changing gender role behaviors of south Asian men in different gender regimes: A comparative study of COVID-19 lockdown in Pakistan and Germany. J Public Aff. 2021;e2797:1–14.

[6] Nyashanu M, Simbanegavi P, Gibson L (2020). Exploring the impact of COVID-19 pandemic lockdown on informal settlements in Tshwane Gauteng Province, South Africa. Global Public Health.

[7] Casale D, Shepherd D. The gendered effects of the ongoing lockdown and school closures in South Africa: Evidence from NIDS-CRAM waves 1 and 2: Department of Economics, University of Stellenbosch; 2020.

[8] John N, Casey SE, Carino G, McGovern T (2020). Lessons Never Learned: Crisis and gender-based violence. Dev world bio.

[9] Usher K, Bhullar N, Durkin J, Gyamfi N, Jackson D (2020). Family violence and COVID-19: Increased vulnerability and reduced options for support. Int j men health nur.

[10] Uzobo E, Ayinmoro AD. Trapped between two pandemics: domestic violence cases under COVID-19 pandemic lockdown: a scoping review. Int Q Community Health Educ. 2021;74:1–10. 10.1177/0272684X211022121 PMC8193047 34102910

[11] Peterman A, Potts A, O’Donnell M, Thompson K, Shah N, Oertelt-Prigione S, et al. Pandemics and violence against women and children. Washington, DC: Center for Global Development, 2020.

[12] Women U. Impact of COVID-19 on violence against women and girls and service provision: UN Women rapid assessment and findings. New York City United Nations Entity for Gender Equality and the Empowerment of Women (UN Women), 2021 21 April 2021. Report No.

[13] Dahal M, Khanal P, Maharjan S, Panthi B, Nepal S (2020). Mitigating violence against women and young girls during COVID-19 induced lockdown in Nepal: a wake-up call. Global health.

[14] Bhatia A, Fabbri C, Cerna-Turoff I, Tanton C, Knight L, Turner E (2020). COVID-19 response measures and violence against children. Bull World Health Org.

[15] Brooks SK, Webster RK, Smith LE, Woodland L, Wessely S, Greenberg N (2020). The psychological impact of quarantine and how to reduce it: rapid review of the evidence. Lancet..

[16] Hamadani JD, Hasan MI, Baldi AJ, Hossain SJ, Shiraji S, Bhuiyan MSA (2020). Immediate impact of stay-at-home orders to control COVID-19 transmission on socioeconomic conditions, food insecurity, mental health, and intimate partner violence in Bangladeshi women and their families: an interrupted time series. Lancet Global Health.

[17] Buller AM, Hidrobo M, Peterman A, Heise L (2016). The way to a man’s heart is through his stomach?: a mixed methods study on causal mechanisms through which cash and in-kind food transfers decreased intimate partner violence. BMC pub health.

[18] Cluver LD, Orkin FM, Meinck F, Boyes ME, Sherr L (2016). Structural drivers and social protection: mechanisms of HIV risk and HIV prevention for South African adolescents. J Int AIDS Soc.

[19] Lawson M, Piel MH, Simon M (2020). Child maltreatment during the COVID-19 pandemic: consequences of parental job loss on psychological and physical abuse towards children. Child abuse negl..

[20] Griffith AK. Parental burnout and child maltreatment during the COVID-19 pandemic. J Fam Violence. 2020;35(5):1–7. 10.1007/s10896-020-00172-2 PMC7311181 32836736

[21] Republic of South Africa. National Strategic Plan on Gender-Based Violence and Femicide. Department of Women, Youth and Persons with Disabilities. Pretoria: Government printers. 2020. p. 1–128.

[22] Jewkes R, Abrahams N, Mathews S, Seedat M, Van Niekerk A, Suffla S, et al. Preventing rape and violence in South Africa: Call for leadership in a new agenda for action. MRC Policy brief. 2009;2.

[23] Machisa M, Jewkes R, Lowe-Morna C, Rama K (2011). The war at home.

[24] Brodie N. Femicide in South Africa Cape Town, South Africa: Kwela Books; 2020. p. 1– 273.

[25] Artz L, Burton P, Ward CL, Leoschut L, Phyfer J, Kassanjee R (2016). Optimus Study South Africa: Technical Report. Sexual victimisation of children in South Africa Final report of the Optimus Foundation Study.

[26] Republic of South Africa. General Household Survey 2020. Statistics South Africa. Pretoria: Government printers. 2020. p. 1–82.

[27] Republic of South Africa. Income dynamics and poverty status of households in South Africa. Statistics South Africa. Pretoria: Government printers. 2012. p. 1–68.

[28] Braun V, Clarke V (2006). Using thematic analysis in psychology. Qual res psych.

[29] Nowell LS, Norris JM, White DE, Moules NJ (2017). Thematic Analysis: Striving to Meet the Trustworthiness Criteria. Int J Qual Meth.

[30] Piquero AR, Jennings WG, Jemison E, Kaukinen C, Knaul FM. Evidence from a systematic review and meta-analysis: Domestic Violence during the COVID-19 Pandemic. J Crim Justice. 2021;74:1–10. 10.1016/j.jcrimjus.2021.101806 PMC9582712 36281275

[31] Casale D, Posel D. Gender and the early effects of the Covid-19 crisis in the paid and unpaid economies in South Africa. National Income Dynamics (NIDS)-Coronavirus Rapid Mobile Survey (CRAM) Wave. 2020;1.

[32] Köhler T, Bhorat H. COVID-19, Social Protection and the Labour Market in South Africa: Are Social Grants being Targeted at the most Vulnerable? 2020.

[33] Hofman K, Madhi S (2020). The unanticipated costs of COVID-19 to South Africa’s quadruple disease burden. SAMJ: South African Med J.

[34] Stiegler N, Bouchard J-P, editors. South Africa: Challenges and successes of the COVID-19 lockdown. Annales Médico-psychologiques, revue psychiatrique; 2020: Elsevier. 10.1016/j.amp.2020.05.006 PMC7250766 32836300

[35] Wills G, Van der Berg S, Patel L, Mpeta B. Household Resource Flows and Food Poverty During South Africa’s Lockdown: Short-term Policy Implications for Three Channels of Social Protection: Department of Economics, University of Stellenbosch; 2020.

[36] Human Science Research Council. COVID-19 survey findings. Pretoria, South Africa: Human Science Research Council; 2020. p. 1–22.

[37] Diamond-Smith N, Conroy AA, Tsai AC, Nekkanti M, Weiser SD (2019). Food insecurity and intimate partner violence among married women in Nepal. J global health..

[38] Hatcher AM, Stöckl H, McBride R-S, Khumalo M, Christofides N (2019). Pathways from food insecurity to intimate partner violence perpetration among peri-urban men in South Africa. American journal of preventive medicine.

[39] Hatcher AM, Weiser SD, Cohen CR, Hagey J, Weke E, Burger R, et al. Food insecurity and intimate partner violence among HIV-positive individuals in rural Kenya. Am J Prev Med. 2020;60(4)563–8. 10.1016/j.amepre.2020.06.025 PMC7987870 33012622

[40] Fulu E, Jewkes R, Roselli T, Garcia-Moreno C. Prevalence and factors associated with male perpetration of intimate partner violence: findings from the UN Multi-country Cross-sectional Study on Men and Violence in Asia and the Pacific. Lancet Global Health. 2013;1(4):187–207. 10.1016/S2214-109X(13)70074-3 25104345

[41] Izugbara CO (2015). ‘Life is not designed to be easy for men’: Masculinity and poverty among urban marginalized Kenyan men. Gender Issues.

[42] Anderson KL, Umberson D (2001). Gendering violence: Masculinity and power in men’s accounts of domestic violence. Gender & society.

[43] Cardoso LF, Gupta J, Shuman S, Cole H, Kpebo D, Falb KL (2016). What factors contribute to intimate partner violence against women in urban, conflict-affected settings? Qualitative findings from Abidjan, Cote d’Ivoire. Journal of Urban Health.

[44] Berke DS, Reidy DE, Gentile B, Zeichner A (2019). Masculine discrepancy stress, emotion-regulation difficulties, and intimate partner violence. Journal of interpersonal violence.

[45] Reidy DE, Berke DS, Gentile B, Zeichner A (2014). Man enough? Masculine discrepancy stress and intimate partner violence. Personality and individual differences.

[46] Sikweyiya Y, Addo-Lartey AA, Alangea DO, Dako-Gyeke P, Chirwa ED, Coker-Appiah D (2020). Patriarchy and gender-inequitable attitudes as drivers of intimate partner violence against women in the central region of Ghana. BMC public health.

[47] Pereda N, Díaz-Faes DA (2020). Family violence against children in the wake of COVID-19 pandemic: a review of current perspectives and risk factors. Child and adolescent psychiatry and mental health.

[48] Fontanesi L, Marchetti D, Mazza C, Di Giandomenico S, Roma P, Verrocchio MC (2020). The effect of the COVID-19 lockdown on parents: A call to adopt urgent measures. Psychological Trauma: Theory, Research, Practice, and Policy..

[49] Achterberg M, Dobbelaar S, Boer OD, Crone E. Home lockdown: Bloom or Boom? Perceived stress as mediator for longitudinal effects of the COVID-19 lockdown on wellbeing of parents and children. 2020. 10.1038/s41598-021-81720-8 PMC7859207 33536464

[50] Patwardhan I, Hurley KD, Thompson RW, Mason WA, Ringle JL (2017). Child maltreatment as a function of cumulative family risk: Findings from the intensive family preservation program. Child Abuse Neglect.

[51] Evans S, Mikocka-Walus A, Klas A, Olive L, Sciberras E, Karantzas G (2020). From ‘It has stopped our lives’ to ‘Spending more time together has strengthened bonds’: The varied experiences of Australian families during COVID-19. Front psych.

[52] Opdenakker R (2006). editor Advantages and disadvantages of four interview techniques in qualitative research.

[53] Mealer M, Jones J (2014). Methodological and ethical issues related to qualitative telephone interviews on sensitive topics. Nur res..

[54] Pickard MD, Roster CA, Chen Y (2016). Revealing sensitive information in personal interviews: Is self-disclosure easier with humans or avatars and under what conditions?. Comp Hum Behav.

